# Nucleotide Diversity of Maize *ZmBT1* Gene and Association with Starch Physicochemical Properties

**DOI:** 10.1371/journal.pone.0103627

**Published:** 2014-08-01

**Authors:** Shuhui Xu, Zefeng Yang, Enying Zhang, Ying Jiang, Liang Pan, Qing Chen, Zhengwen Xie, Chenwu Xu

**Affiliations:** 1 Jiangsu Key Laboratory of Crop Genetics and Physiology/Co-Innovation Center for Modern Production Technology of Grain Crops, Key Laboratory of Plant Functional Genomics of the Ministry of Education, Yangzhou University, Yangzhou, China; 2 College of Agronomy and Plant Protection, Qingdao Agricultural University, Qingdao, Shandong, China; Nanjing Agricultural University, China

## Abstract

Cereal Brittle1 protein has been demonstrated to be involved in the ADP-Glc transport into endosperm plastids, and plays vital roles in the biosynthesis of starch. In this study, the genomic sequences of the *ZmBT1* gene in 80 elite maize inbred lines were obtained, and the nucleotide polymorphisms and haplotype diversity were detected. A total of 30 variants, including 22 SNPs and 8 indels, were detected from the full sequences of this gene. Among these polymorphic sites, 9 SNPs and 2 indels were found to be located in the coding region. The polymorphisms of CDS sequences classified the maize *ZmBT1* gene into 6 haplotypes, which encode 6 different ZmBT1 proteins. Neutrality tests revealed a decrease in population size and/or balancing selection on the maize *ZmBT1* locus. To detect the association between sequence variations of this gene and the starch physicochemical properties, 7 pasting and 4 gelatinization traits of starch were measured for the tested inbred lines using rapid visco analyzer (RVA) and differential scanning calorimeter (DSC), respectively. The result of association analysis revealed that an indel in the coding region was significantly associated with the phenotypic variation of starch gelatinization enthalpy.

## Introduction

Starch or amylum is a carbohydrate consisting of a large number of glucose units joined by glycosidic bonds. Starch rich crops are the main source of dietary energy for the world's population. It has been believed that plant species share the evolutionarily conserved pathway of starch biosynthesis starting from the carbon dioxide fixation, followed by transitory starch degradation, sucrose synthesis, and starch synthesis in the storage organs [1]. Four classes of enzymes are included in the starch biosynthesis pathway, they are ADP-glucose pyrophosphorylase (AGPase), starch synthase (SS), starch branching enzyme (SBE), and starch debranching enzyme (DBE) [2], [3]. Among them, AGPase catalyzes the first committed and rate-limiting step in this pathway, and plays vital role in the biosynthesis of starch [4]. Under the catalyzing of AGPase, ADP-glucose (ADP-Glc) is synthesized in the cytosol of cereal endosperms as the main precursor for starch synthesis and has to be subsequently imported into the storage plastids [4]. Because of the importance of cereals in the production of storage starches for human diet and other industrial usage, the activity of the ADP-Glc transporter have gained many attentions as a key component of the starch biosynthesis pathway [5].

One of the ADP-Glc transporters demonstrated clearly in cereal is the protein Brittle1. Brittle1 proteins are plant nucleotide transporters involved in the mitochondrial carrier family (MCF) [6]. The proteins in MCF family transports nucleotides, amino acids, inorganic ions, fatty acids, keto acids and cofactors across the mitochondrial membrane [7]. Physiological researches on the maize *Brittle1* mutant have revealed that ZmBT1 (*Zea mays* Brittle1 protein) was involved in the ADP-Glc transport into endosperm plastids, and played critical roles in the biosynthesis of starch [4], [8], [9]. The maize endosperm with *bt1* mutant is severely reduced in starch content, which results in kernels with a collapsed angular appearance at maturity [4]. The amyloplasts from young kernels isolated from endosperms with *bt1* mutant were only 25% as active in ADP-Glc uptake and conversion to starch as amyloplasts from normal and mutant maize endosperms, suggesting that ZmBT1 is involved in the transport of ADP-Glc into maize endosperm plastids [8], [10]. The researches in other cereals also revealed that the homologs of BT1 protein possessed the ability in transporting ADP-Glc. For example, the barley *lys5* mutant, a homolog of *BT1* (*HvNST1*), show a reduced capacity for ADP-Glc uptake by isolated endosperm amyloplasts [5], [11]. However, the BT1 proteins in dicots, such as AtBT1 in *Arabidosis* and StBT1 in *Solanum tuberosum* do not transport ADP-Glc, but instead transport AMP, ADP and ATP in a unidirectional mode [12].

Maize (*Zea mays* L.) is one of the most important grown cereals in the world. It provides staple food to many populations, as well as a major nutrient source for animal feed. In addition, benefitting from its unique character such as low pasting temperature and slow tendency of retrogradation, maize starch is one of the important raw materials for industrial production of food. The pasting properties of maize starch will enormously affect fabrication property, flavor characteristics and keeping in storage. Recently, the RVA profile of starch paste viscosity was widely employed to evaluate the quality of cereal crops, because this method requires only a small size and the procedure is easy to perform [13], [14]. Starch gelatinization, one of the most important and unique properties, refers to the process of the disruption of granular structure causing starch molecules to dissolve in water [15]. The gelatinization properties of starch are the most important indexes in many food modification including cooking, baking and extruding starch-based foods [16]. Although the physiological roles on the starch biosynthesis of the maize *ZmBT1* gene has been illustrated, the effect of this gene in the formation of maize starch pasting and gelatinization properties reminds unknown. Moreover, there is no association analysis between the nucleotide polymorphisms of the maize *ZmBT1* gene and the physicochemical properties of maize starch. In this work, we analyzed the nucleotide polymorphism of maize *ZmBT1* locus, and investigate the association between the sequence polymorphisms of the maize *ZmBT1* gene and some starch pasting and gelatinization properties.

## Materials and Methods

### Plant materials and sequencing the maize *ZmBT1* gene

A total of 80 elite maize inbred lines were used in this study. These inbred lines were also the representative lines, including temperate germplasm from 5 heterotic groups, tropic and waxy germplasm (Table 1). They represented most of the genetic diversity available to breeding and research programs in China. In addition, some germplasm introduced from other countries were also included in this study. The inbred lines were grown in two-row plots with an randomized block design of two repetitions in a natural environment during 2012 in Sanya, Hainan province. The mature seeds for each inbred lines were harvested in bulk for phenotypic data analysis.

**Table 1:** List of the 80 maize inbred lines used in this study.

No.	Inbred Line	Origin and Pedigree	No.	Inbred Line	Origin and Pedigree
1	QH19612	Derived from Huangzaosi	41	6819	Derived from P78599
2	Chang7-2	(Huangzaosi×Wei95) ×S901	42	Dan988	Derived from P78599
3	LX9801	Ye502×H21	43	319B	Derived from P78599
4	107	Dekalb×L80	44	Qi319	Derived from P78599
5	k12	Huangzaosi×Huaichun	45	Qi318	Derived from P78599
6	nx335	NF358×PH4CV	46	Shen137	Derived from P6JK111
7	Ji853	(Huangzaosi×Zi330) ×Huangzaosi	47	11099	Tropical germplasm
8	412	Mo17×Jing09	48	suwan	Tropical germplasm
9	Za107	Dekalb×L80	49	11118	Tropical germplasm
10	Huangzaosi	Derived from Sipingtou	50	11200	Tropical germplasm
11	502	Huangzaosi×Dan340	51	10533-1	Tropical germplasm
12	Luyuan92	Yuanqi122×1137	52	RCML15	Tropical germplasm
13	10168	Derived from 5003	53	RBS11	Tropical germplasm
14	Dan598	(Dan340×Danhuang11) ×(Danhuang02×599)	54	Q52	Derived from an USA crossbreed
15	Zong3	Zi330×Lv28	55	KWS456	Derived from KWS
16	4CV	Derived from Mo17	56	QF-11	Unknown
17	E28	(A619Ht1×Lvjiukuan) ×Lvjiukuan	57	QF01	Derived from French germplasm
18	S122	(YeH201×Dan340) ×Dan340	58	JND-1	Derived from a Canadian germplasm
19	Dan340	Baigulv9×Pod corn	59	BJ-2	Derived from Mo17
20	Danhuang25	Derived from P78599	60	QF02	Derived from French germplasm
21	JH3372	Shen5003×Zi330	61	QDM01	Derived from KWS
22	Dan99	Derived from Lvdahonggu35	62	M1	Unknown
23	Qi232	Unknown	63	WT262	Derived from KWS
24	OH43	OH40b×W8	64	WT26	Derived from KWS
25	MO17	C103×187-2	65	Y53	Unknown
26	8112	P3382×P3147	66	BJ-5	Derived from Mo17
27	K8112	P3382×P3147	67	QZN01F	Waxy germplasm
28	Wu314	(302D×Huangbaoli) ×Huangzaosi	68	QKN01M	Waxy germplasm
29	4866	7922×Ye478	69	QDT01	Waxy germplasm
30	3189	U8112×Shen5003	70	QLY01Z	Waxy germplasm
31	Tie9206	Tie8706×Tie8708	71	QJKN20F	Waxy germplasm
32	Benyu15	Unknown	72	QZN12	Waxy germplasm
33	478Xuan	U8112×Shen5003	73	QKN01F	Waxy germplasm
34	Zheng58	Derived from Ye478	74	QBN48	Waxy germplasm
35	7922	Derived from P3382	75	QBN029	Waxy germplasm
36	178	P78599×Mo17	76	BEM	Waxy germplasm
37	QP1721	Derived from P78599	77	QBN02	Waxy germplasm
38	Exhan	Derived from P78599	78	QBN3186	Waxy germplasm
39	xy35	Derived from Xianyu335	79	M1132	Waxy germplasm
40	P138	Derived from P78599	80	QZN012	Waxy germplasm

Young plant leaves were collected at the four-leaf stage for each accession and stored at −80°C until genomic DNA extraction. Genomic DNA was extracted from the frozen young leaves of the 80 inbred lines using CTAB (cetyl trimethyl ammonium bromide) method [17] according to the modified protocol. The sequences of the *ZmBT1* gene in the tested inbred lines were sequenced using the target sequence capture sequencing technology on the NimbleGen platform [18] by BGI Life Tech Co., Ltd.. The genomic sequence and position of the maize *ZmBT1* gene (GRMZM2G144081) of the inbred line B73 were used as the reference sequences for target sequence capture.

### Measurement of maize starch pasting and gelatinization properties

The pasting properties were measured using a rapid visco analyser (RVA) (Model No. RVA-3D, Newport Scientific, Sydney, Australia). A total of 3-g starch from each inbred line was dispersed in 25 ml of distilled water in the viscometer test canister. The sequential temperature curve for a 12.5 min test was as follows: (1) incubate at 50°C for 1.0 min; (2) increase to 95°C; (3) keep at 95°C for 2.5 min; (4) cool down to 50°C; and (5) hold at 50°C for 1.4 min. The viscosity was evaluated using a constant paddle rotation of 160 rpm. Viscosity values were recorded in centipose (cp).

The gelatinization properties of mazie starches were analyzed using a differential scanning calorimeter DSC 200F3 Maia (Netzsch, Germany). Starch samples (5 mg, dried starch basis) were precisely weighed in the sample pans, mixed with distilled water (10 µl), and sealed. The heating rate was at 10°C per min over the temperature range of 20–100°C. The gelatinization properties were recorded with a thermal analysis data station equipped in DSC.

### Sequence analysis

Multiple sequence alignment of the maize *ZmBT1* gene was performed using Clustal X and was further edited manually. The software DNASP 5.0 [19], [20] was used to analyze sequence nucleotide polymorphism and allelic diversities. Two parameters of nucleotide diversity, 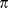 and 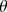
_,_ were estimated. Where 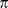 is the average number of nucleotide differences per site between any two DNA sequences, and 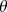 is derived from the total number of segregating sites and corrected for sampling size. Tajima's D [21] and Fu and Li's D* and F* [22] statistical tests were used to test the evidence of neutral evolution within the selected population and each defined region. The minimum number of recombination events [23] was estimated in the period of evolution of *ZmBT1* gene among the tested inbred lines. The linkage disequilibrium (LD) between any two polymorphic sites were estimated using TASSEL v3.0 [24]. In addition, the decay of LD with physical distance in *ZmBT* gene was evaluated by regression analysis (PROC NLIN and REG in SAS software). The regression models, including linear, loglinear, exponential, power and Remington's models [25], were used in this study.

### Population structure and association analysis

Population structure is a major bias factor leading to false-positive associations. To alleviate the effect of population structure, all inbred lines were genotyped with the SNP chips contained 3,072 random SNP markers evenly covering the maize genome. These SNP markers were selected from 49,585 SNP markers used by recently reported chips [26]. SNP genotyping was performed via the GoldenGate assay at the National Maize Improvement Centre of China, China Agricultural University. The population structure was evaluated by these SNP markers, and the resulting Q-values were obtained from the STRUCTURE program [27]. Five independent runs were performed setting the number of populations (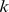) from 2 to 8, burn in time and MCMC (Markov Chain Monte Carlo) replication number both to 100,000, and a model for admixture and correlated allele frequencies. The 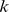 value was determined by LnP(D) in STRUCTURE output and an ad hoc statistic 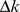 based on the rate of change in LnP(D) between successive 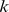. The tests of significant association between the sequence polymorphisms with Minor Allele Frequency (MAF) 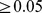 and starch pasting and gelatinization properties in the tested population were performed using the general linear model (GLM) in the TASSEL software v3.0 [24].

## Results

### Nucleotide diversity and selection of the maize *ZmBT1* gene

The position and nucleotide sequences of the maize *ZmBT1* gene in inbred line B73, whose genome has been fully sequenced, were used as the references to capture of the sequences of this gene in 80 inbred lines. Sequence polymorphisms were detected among 80 maize inbred lines across 2,442 bp of sequence, which covers a 520 bp 5′ upstream promoter region, a 624 bp exon_1 region, a 131 bp intron_1 region, a 162 bp exon_2 region, a 128 bp intron_2 region, a 534 bp exon_3 region and a 337 bp 3'-UTR region. Nucleotide substitutions and indels at the *ZmBT1* locus were identified, and the results were summarized in Table S1 and Table 2. From the putative genomic sequences in 80 maize inbred lines, a total of 30 variants were identified, including 22 SNP sites and 8 indels. Among all the SNP sites, only one belongs to singleton variable site, while the other 21 sites belong to parsimony informative sites (Tables 2 and S1). In addition, 2 indels were found to be singleton variations, while the other 6 indels belonged to parsimony variations. For all the 80 inbred lines, the overall nucleotide diversity (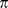) of *ZmBT1* locus was 0.00351. However, we also noticed that the polymorphic sites were unevenly distributed among 7 defined regions of maize *ZmBT1* locus. There is no nucleotide substitution in the regions of intron1, exon2 and intron2. In addition, no indel was found in exon2, while all the other regions possessed at least one indel.

**Table 2:** Summary of parameters for the analysis of nucleotide polymorphisms of the maize gene *ZmBT1.*

Parameters	Promoter	Exon1	Intron1	Exon2	Intron2	Exon3	3'-UTR	Entire region
Total length of amplicons (bp)	520	624	131	162	128	534	337	2442
Number of all sequence variants (SNPs and indels)	8	6	3	0	1	5	7	30
Number of nucleotide substitutions (bp)	7	5	0	0	0	4	6	22
Number of indels	1	1	3	0	1	1	1	8
Number of indel sites	1	3	19	0	7	6	1	37
Average indel length	1	3	6.3333	NAN	7	6	1	4.625
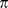	0.00590	0.00407	0	0	0	0.00297	0.00383	0.00351
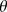	0.00272	0.00163	0	0	0	0.00153	0.00361	0.00185
Tajima's D	2.83337**	3.32754**	NAN	NAN	NAN	1.93935	0.14499	2.71716**
Fu and Li's D*	0.36446	1.05574	NAN	NAN	NAN	0.96034	1.13780	1.37729
Fu and Li's F*	1.39842	2.10430*	NAN	NAN	NAN	1.49149	0.95695	2.23468*

The Tajima's D statistic is a widely used test to identify sequences which do not fit the neutral theory model at equilibrium between mutation and genetic drift. In this analysis, the estimates of Tajima's D in the regions of promoter and exon1 were both statistically higher than 0 at the level of 0.01. In addition, we also noticed that the Tajima's D statistic for the entire region of the maize *ZmBT1* gene was statistically higher than zero. Furthermore, when we combined all three exons, the estimate of Tajima's D was 3.23599, which was also statistically significant at the level of 0.01. These results revealed that low levels of both low and high frequency polymorphisms in maize *ZmBT1* locus, and also indicated a decrease in population size and/or balancing selection. In addition, the estimates of Fu and Li's F* for both coding (2.3416) and entire regions were significant for the *ZmBT1* gene, also suggesting balancing selection on this gene.

### Haplotype diversity of the maize *ZmBT1* gene

According to the full length of the *ZmBT1* gene in the tested 80 maize inbred lines, a total of 11 haplotypes were detected with a haplotype diversity (*Hd*) equal to 0.7734 (Table 3). The tested inbred lines were unbalancedly distributed in these haplotypes. Among the haplotypes identified in this analysis, 6 contained only one inbred line. The most frequent haplotype was Hap_1, which contained 29 inbred lines. In addition, we also noticed that four frequent haplotypes, including Hap_1–4, contained 90% of the tested inbred lines.

**Table 3:** The distribution of haplotypes of *ZmBT1* gene in 80 inbred lines using both the full-length sequence and coding regions.

Haplotype	Number	CDS haplotype	Inbred line
Hap_1	29	CDS_Hap_1	Wu314, Dan988, QH19612, Exhan, Dan99, QZN12, M1132, 502, Qi319, QF01, QBN029, JND-1, QDT01, QKN01F, P138, Luyuan92, 178, Shen137, 6819, Chang7-2, QDM01, WT262, LX9801, 107, JH3372, 11200, BJ-5, Danhuang25, 412
Hap_2	16	CDS_Hap_2	nx335, RBS11, QJKN20F, xy35, M1, 11099, 7922, Tie9206, S122, QF02, Y53, 4CV, WT26, Mo17, BJ-2, BEM
Hap_3	18	CDS_Hap_3	QLY01Z, RCML15, QBN02, E28, Q52, Qi232, K8112, Benyu15, 8112, QZN01F, 11118, 4866, Za107, Dan340, 319B, QF-11, Zong3, Zheng58
Hap_4	9	CDS_Hap_4	QZN012, QBN48, KWS456, QBN3186, 10533-1, 3189, Qi318, 10168, 478Xuan
Hap_5	1	CDS_Hap_1	OH43
Hap_6	1	CDS_Hap_5	QP1721
Hap_7	2	CDS_Hap_6	QKN01M, Dan598
Hap_8	1	CDS_Hap_1	Huangzaosi
Hap_9	1	CDS_Hap_5	k12
Hap_10	1	CDS_Hap_1	suwan
Hap_11	1	CDS_Hap_6	Ji853

In the coding region of the maize *ZmBT1* gene, a total of 9 SNPs were detected. In addition, 2 indels were also identified in the coding regions. When we used the coding sequences to identify the hapotype diversity, a total of 6 haplotypes were identified for these 80 inbred lines (Table 3) with a *Hd* equal to 0.7440. Each of the haplotypes defined by coding sequences of *ZmBT1* gene contained at least 2 inbred lines. The most frequent CDS haplotype was CDS_Hap_1, which contained 32 inbred lines.

**Figure 1:**
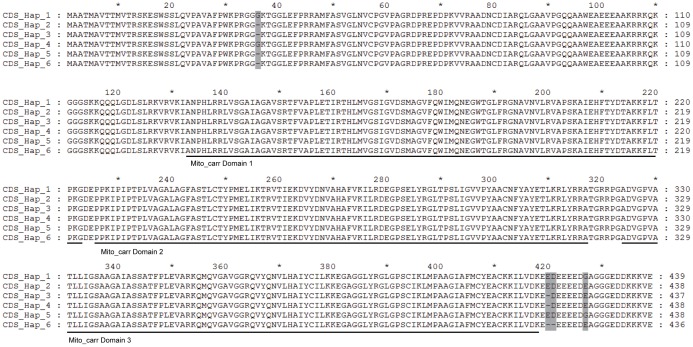
Sequence alignment of maize ZmBT1 proteins encoded by different CDS haplotypes. The haplotypes defined by the coding sequences of the maize *ZmBT1* gene were used as the sequence names. Polymorphisms from inferred amino acids were indicated by boxes. Three mitochondrial carrier protein domains were indexed by lines.

Among the SNPs detected in the coding regions, only one belonged to the nonsynonymous site which could cause the replacement change of amino acid sequences. In addition, the variation of 2 indels could also result in the change of ZmBT1 protein. Both of these two indels covered 3 or 6 nucleotides, which will not result in frame shift during translation. The indel7 contained three types, including no deletions, 3 and 6 nucleotides deletions. When we translated the CDS into amino acid sequences, 6 types of ZmBT1 protein sequences were found to be encoded by these inbred lines (Fig. 1). Three evolutionarily conserved mitochondrial carrier protein domains (Mito_carr, PF00153) were detected in maize ZmBT1 protein using the tool of Pfam. However, None of the four variants of amino acid caused by two indels and one nonsynonymous SNP was located in the regions of these three domains.

### Linkage disequilibrium and recombination events

**Figure 2:**
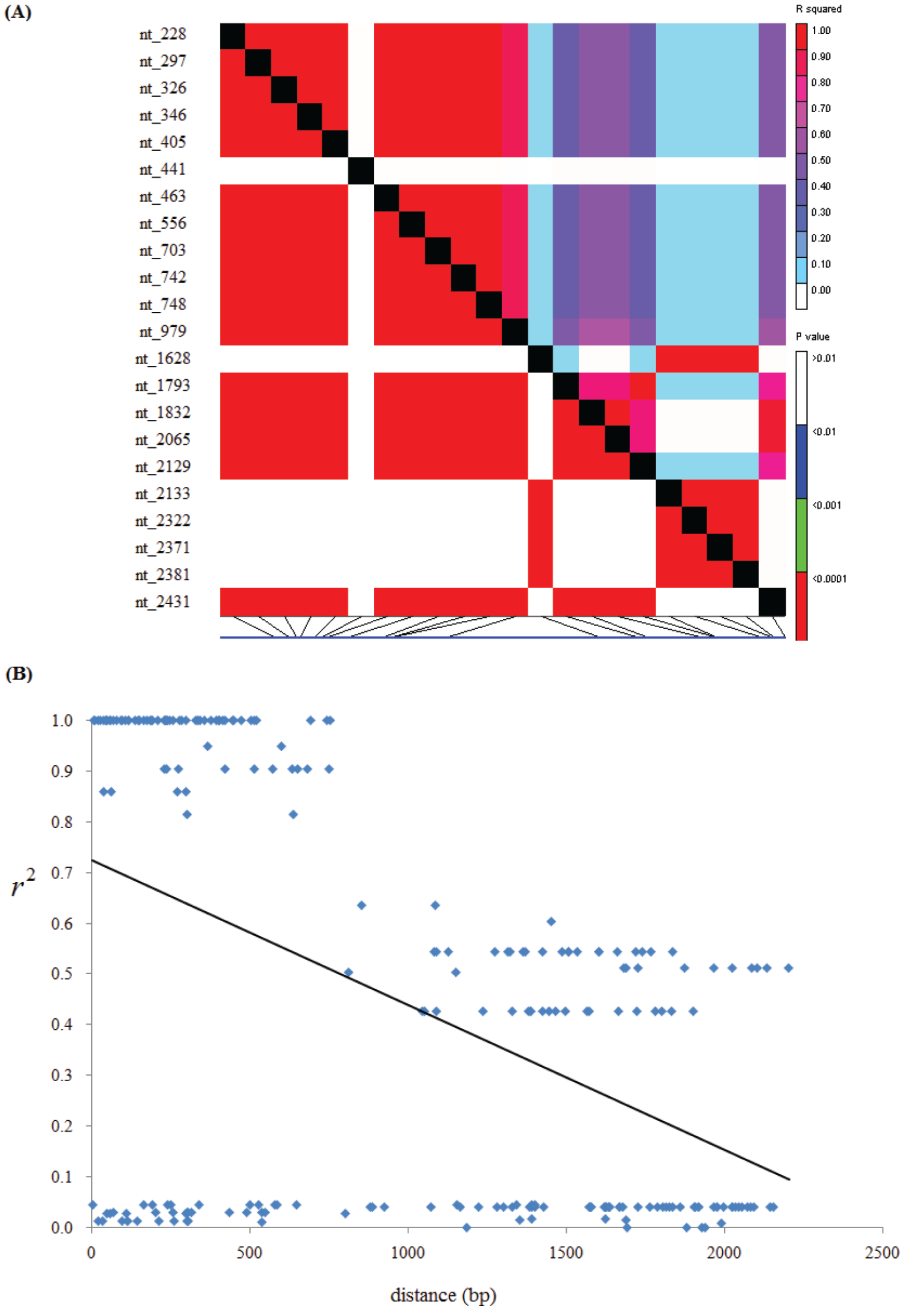
LD patterns across the whole locus of *ZmBT1*. (A) LD between pairs of *ZmBT1* sequence polymorphic sites. (B) Decay of LD between pairs of *ZmBT1* sequence informative polymorphisms. The linear regression coefficient 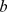 is −0.00029.

Linkage disequilibrium was investigated between pairwise segregating sites in order to predict the expected resolution and marker density needed for candidate-gene association mapping. In this analysis, all the SNPs identified in maize *ZmBT1* gene and the values of 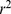 were used and the result revealed that more than half of the pairs between any two polymorphic sites of maize *ZmBT1* gene (130 out of 231 for the tested LD are significant at 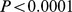) showed significant linkage disequilibrium (LD). To test the decay of LD with increasing physical distance, some regression equations, including linear, loglinear, exponential, power and Remington's models, were estimated. In this analysis, the linear regression model was selected to fit the data, because this model possessed the highest coefficient of determination. Our result revealed that the LD decayed rapidly with increasing physical distance. The predicted value of 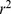 declined to 0.1 within 2184 bp at *ZmBT1* locus (Fig. 2).

The polymorphic sites in the entire *Zmisa2* locus were used to detect the evidence of recombination. The patterns of the polymorphisms identified in inbred lines surveyed in this study indicated the history of recombination at *ZmBT1* locus, which contributed to the haplotype diversity and the decay of LD. However, only one recombination event has been detected according to the algorithm of Hudson and Kaplan for minimum number of recombination events, and the recombination has been detected between sites 979–1793 bp.

### The phenotypic variations and association analysis

Pasting properties of various corn starches measured by RVA, including PV, TV, BD, FV, SB, PT and PTP have been summarized in Table 4. The gelatinization temperatures (onset, 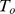; peak, 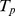; and conclusion, 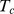) and enthalpy of gelatinization (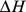), for maize starches from different inbred lines, measured using DSC are also presented. Significant difference in all the pasting and gelatinization properties among different maize inbred lines was observed through one-way ANOVA. These results suggest that the 80 inbred lines used in this study are representative in terms of maize quality and are qualified for association analysis.

**Table 4:** The descriptive statistics and the results of one-way ANOVA for 7 starch pasting and 4 gelatinization properties of 80 maize inbred lines.

Parameter	PV	TV	BD	FV	SB	PT	PTP	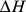	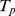	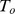	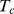
Mean	1317.013	926.856	390.156	2575.488	1258.563	5.336	72.759	6.225	75.591	70.912	81.266
Standard deviation	405.135	250.758	216.227	841.476	593.455	0.391	2.641	1.008	1.037	1.219	1.989
Minimum	577.5	422	38	892.5	−161.5	4.535	69.15	4.244	73.25	67.65	70.25
Maximum	2321.5	1406.5	924	4066	2160.5	7.165	93.075	8.1	77.5	73.5	83.9
*F*	130.35	77.74	99.17	258.13	235.29	46.07	69.43	5.62	11.28	12.82	62.73
*P*	<0.0001	<0.0001	<0.0001	<0.0001	<0.0001	<0.0001	<0.0001	<0.0001	<0.0001	<0.0001	<0.0001

To explore the relationship among 11 starch pasting and gelatinization properties, the pairwise correlation analysis was performed, and the Pearson correlation coefficients (*r*) between any two parameters were obtained (Table 5). Interestingly, the significant correlations were found between any two pasting parameters, and only 5 pairwise correlations, including PT/PV, PT/TV, PT/FV, PT/SB and SB/PTP did not reach the significant level. Among the 6 pairwise correlations for gelatinization properties, only the pairs of 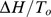 and 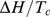 showed no significance. In addition, the correlations between the pasting and gelatinization properties were also investigated, and the results revealed that 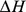 showed significant correlations with PV, TV, BD, PT and PTP, 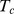 with PV and BD, and 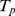 with BD. These results suggested that potentially different genetic mechanisms were responsible for these starch viscosity properties.

**Table 5:** The pairwise correlation analysis among 7 pasting and 4 gelatinization properties of maize starch.

	PV	TV	BD	FV	SB	PT	PTP	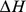	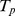	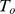
TV	0.887**									
BD	0.845**	0.503**								
FV	0.762**	0.665**	0.657**							
SB	0.398**	0.337**	0.355**	0.897**						
PT	−0.212	−0.046	−0.343**	0.049	0.215					
PTP	−0.422**	−0.421**	−0.302**	−0.259*	−0.078	0.645**				
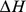	0.353**	0.371**	0.232*	0.065	−0.149	−0.558**	−0.593**			
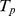	−0.157	−0.005	−0.290**	−0.218	−0.203	−0.094	−0.024	0.239*		
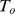	0.018	0.093	−0.073	0.084	0.106	−0.069	−0.032	0.181	0.851**	
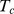	−0.250*	−0.148	−0.298**	−0.214	−0.133	−0.141	−0.084	0.103	0.468**	0.342**

GLM of association analysis that controlled the effects of population structure was used to identify relative association of 11 starch pasting and gelatinization properties and genotype variants in maize *ZmBT1* gene. All nucleotide polymorphisms, including SNPs and indels, with a frequency of more than 0.05 of the rare alleles were considered in the association analysis of phenotype-genotype in both genes. Only one variant (indel7 in exon2) in maize *ZmBT1* gene showed significant association with 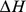, while all the other variants had no association with starch pasting and gelatinization properties. Indel7 can cause a deletion of glutamic acid (E) in 27 inbreds or a deletion of glutamic and asparagic acids (ED) in 3 inbreds. Because the latter possessed a frequency of lower than 0.05 in the tested population, it was not used in the association analysis. According to the result of association analysis, indel7 explained 9.26% of the phenotypic variant of starch 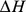. The mean value of 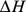 was 6.091 with a standard deviation 1.087 for the alleles carrying the deletion of glutamic acid (E) in protein product, and this value was statistically lower than those without deletion (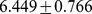) based on independent samples *t* test (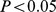).

## Discussion

The abundant genetic variations enable plant breeders to create novel plant gene combinations and select crop varieties more suited to the needs of diverse agricultural systems. The analysis of the genetic diversity for crop functional genes is critical for understanding the genetic background of phenotypic variation, and in turn will provide great help for crop improvement [28], [29]. In this study, 30 variants, including 22 SNPs and 8 indels, were identified in the full-length sequence of the maize *ZmBT1* gene. Among these SNPs, 9 were found in the coding region, one of which were nonsynonymous and the others were synonymous. In addition, there were two indels in the coding region of this gene. The nonsynonymous SNPs and indels in the coding region would result in the changes of protein product. The SNP sites and indels in the coding region also classified the tested inbred lines into 6 haplotypes, which encode 6 deferring ZmBT1 proteins. However, lower frequency of variant was found in the intron regions of this gene. Particularly, none of the SNPs was identified in two introns of this gene. This may be the result of that the intron regions in this gene are much shorter than the coding region.

In this study, significantly positive statistics were obtained for promoter, coding and entire regions of the maize *ZmBT1* gene through Tajima's neutrality tests. Thus, a decrease in population size and/or balancing selection was suggested for this gene. Balancing selection refers to a number of selective processes by which multiple alleles are actively maintained in the gene pool of a population at frequencies above that of gene mutation. Balancing selection usually happens when the heterozygotes for the alleles under consideration have a higher adaptive value than the homozygote [30]. Thus, potential high heterozygosity at the *ZmBT1* locus in the tested population is suggested.

LD is the non-random association between allelic polymorphisms at two loci. It was suggested that recombination and selection were the main determinants of LD [31]. Maize is an outcrossing crop with extensive morphological variation, genetic diversity and high effective frequency of recombination [32]. Recent researches revealed that the rapid breakdown of LD in diverse sets of maize germplasm [25], [33]. In this analysis, we found that the decay of LD in the maize *ZmBT1* locus was slower than the expected value. This may be the result of low frequency of recombination in this gene, because only one recombination event was detected in the *ZmBT1* gene.

Gelatinization temperature and enthalpy of maize starch plays an important role in grain quality. The enthalpy of gelatinization gives an overall measure of crystallinity and may be indicative of the loss of molecular order within the granule [34]. Previously, some genes in starch biosynthesis pathway were found to affect the phenotypic variation of starch gelatinization properties. Based on the strategy quantitative trait loci (QTL) mapping, Tan et al. demonstrated that the *Wx* gene and two loci including starch-branching enzyme (SBE) genes in rice controlled the starch gelatinization properties [34]. According to the results of association analysis, the sequence variations of rice genes *wx*, *SSI*, and *SSII-3* were found to be associated with gelatinization properties 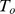, 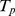 and 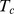 in waxy rice. In addition, the enthalpy of gelatinization (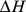) of rice starch is controlled by *wx* and *SSII-3* [35]. The cereal protein BT1 is involved in the ADP-Glc transport into endosperm plastids, and played vital roles in the biosynthesis of starch. In this study, we showed that the maize *ZmBT1* gene possessed abundant nucleotide polymorphism. Further evidence based on association with pasting and gelatinization properties revealed that an indel in coding regions of this gene was associated with gelatinization enthalpy (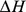) of maize starch. Although 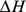 showed correlations with other pasting and gelatinization properties, no association was found between the polymorphic sites and these traits. In addition, the formation of starch pasting and gelatinization properties is a complex process, and all these properties are quantitative traits influenced by multiple genes. Thus, these results obtained needs further verification owing to that only one gene *ZmBT1* in the starch biosynthesis was used.

## Supporting Information

Table S1 The positions of nucleotide polymorphism of *ZmBT1* gene among 80 maize inbred lines.
(XLSX)

## References

[1] Saithong T, Rongsirikul O, Kalapanulak S, Chiewchankaset P, Siriwat W, et al (2013) Starch biosynthesis in cassava: a genome-based pathway reconstruction and its exploitation in data integration. BMC Syst Biol 7: 75. 23938102 10.1186/1752-0509-7-75 PMC3847483

[2] Yang Z, Wang Y, Xu S, Xu C, Yan C (2013) Molecular evolution and functional divergence of soluble starch synthase genes in cassava (manihot esculenta crantz). Evol Bioinform Online 9: 239–249. 23888108 10.4137/EBO.S11991 PMC3712559

[3] Tian Z, Qian Q, Liu Q, Yan M, Liu X, et al (2009) Allelic diversities in rice starch biosynthesis lead to a diverse array of rice eating and cooking qualities. Proc Natl Acad Sci U S A 106: 21760–21765. 20018713 10.1073/pnas.0912396106 PMC2793318

[4] Kirchberger S, Leroch M, Huynen MA, Wahl M, Neuhaus HE, et al (2007) Molecular and biochemical analysis of the plastidic ADP-glucose transporter (ZmBT1) from Zea mays. J Biol Chem 282: 22481–22491. 17562699 10.1074/jbc.M702484200

[5] Bowsher CG, Scrase-Field EF, Esposito S, Emes MJ, Tetlow IJ (2007) Characterization of ADP-glucose transport across the cereal endosperm amyloplast envelope. J Exp Bot 58: 1321–1332. 17301030 10.1093/jxb/erl297

[6] Bahaji A, Ovecka M, Barany I, Risueno MC, Munoz FJ, et al (2011) Dual targeting to mitochondria and plastids of AtBT1 and ZmBT1, two members of the mitochondrial carrier family. Plant Cell Physiol 52: 597–609. 21330298 10.1093/pcp/pcr019

[7] Laloi M (1999) Plant mitochondrial carriers: an overview. Cell Mol Life Sci 56: 918–944. 11212326 10.1007/s000180050484 PMC11146799

[8] Shannon JC, Pien FM, Cao H, Liu KC (1998) Brittle-1, an adenylate translocator, facilitates transfer of extraplastidial synthesized ADP–glucose into amyloplasts of maize endosperms. Plant Physiol 117: 1235–1252. 9701580 10.1104/pp.117.4.1235 PMC34888

[9] Sullivan TD, Kaneko Y (1995) The maize brittle 1 gene encodes amyloplast membrane polypeptides. Planta 196: 477–484. 7647682 10.1007/BF00203647

[10] Li HM, Sullivan TD, Keegstra K (1992) Information for targeting to the chloroplastic inner envelope membrane is contained in the mature region of the maize Bt1-encoded protein. J Biol Chem 267: 18999–19004. 1527026

[11] Patron NJ, Greber B, Fahy BF, Laurie DA, Parker ML, et al (2004) The lys5 mutations of barley reveal the nature and importance of plastidial ADP-Glc transporters for starch synthesis in cereal endosperm. Plant Physiol 135: 2088–2097. 15299120 10.1104/pp.104.045203 PMC520780

[12] Leroch M, Kirchberger S, Haferkamp I, Wahl M, Neuhaus HE, et al (2005) Identification and characterization of a novel plastidic adenine nucleotide uniporter from Solanum tuberosum. J Biol Chem 280: 17992–18000. 15737999 10.1074/jbc.M412462200

[13] Yan CJ, Tian ZX, Fang YW, Yang YC, Li J, et al (2011) Genetic analysis of starch paste viscosity parameters in glutinous rice (Oryza sativa L.). Theor Appl Genet 122: 63–76. 20737264 10.1007/s00122-010-1423-5

[14] Sanchez T, Dufour D, Moreno IX, Ceballos H (2010) Comparison of pasting and gel stabilities of waxy and normal starches from potato, maize, and rice with those of a novel waxy cassava starch under thermal, chemical, and mechanical stress. J Agric Food Chem 58: 5093–5099. 20356303 10.1021/jf1001606

[15] Ratnayake WS, Jackson DS (2006) Gelatinization and solubility of corn starch during heating in excess water: new insights. J Agric Food Chem 54: 3712–3716. 19127749 10.1021/jf0529114

[16] Hasjim J, Li E, Dhital S (2013) Milling of rice grains: effects of starch/flour structures on gelatinization and pasting properties. Carbohydr Polym 92: 682–690. 23218354 10.1016/j.carbpol.2012.09.023

[17] Fulton TM, Chunwongse J, Tanksley SD (1995) Microprep protocol for extraction of DNA from tomato and other herbaceous plants. Plant Molecular Biology Reporter 13: 207–209.

[18] Asan, Xu Y, Jiang H, Tyler-Smith C, Xue Y, et al (2011) Comprehensive comparison of three commercial human whole-exome capture platforms. Genome Biol 12: R95. 21955857 10.1186/gb-2011-12-9-r95 PMC3308058

[19] Librado P, Rozas J (2009) DnaSP v5: a software for comprehensive analysis of DNA polymorphism data. Bioinformatics 25: 1451–1452. 19346325 10.1093/bioinformatics/btp187

[20] Rozas J (2009) DNA sequence polymorphism analysis using DnaSP. Methods Mol Biol 537: 337–350. 19378153 10.1007/978-1-59745-251-9_17

[21] Tajima F (1989) Statistical method for testing the neutral mutation hypothesis by DNA polymorphism. Genetics 123: 585–595. 2513255 10.1093/genetics/123.3.585 PMC1203831

[22] Fu YX, Li WH (1993) Statistical tests of neutrality of mutations. Genetics 133: 693–709. 8454210 10.1093/genetics/133.3.693 PMC1205353

[23] Hudson RR, Kaplan NL (1985) Statistical properties of the number of recombination events in the history of a sample of DNA sequences. Genetics 111: 147–164. 4029609 10.1093/genetics/111.1.147 PMC1202594

[24] Bradbury PJ, Zhang Z, Kroon DE, Casstevens TM, Ramdoss Y, et al (2007) TASSEL: software for association mapping of complex traits in diverse samples. Bioinformatics 23: 2633–2635. 17586829 10.1093/bioinformatics/btm308

[25] Remington DL, Thornsberry JM, Matsuoka Y, Wilson LM, Whitt SR, et al (2001) Structure of linkage disequilibrium and phenotypic associations in the maize genome. Proc Natl Acad Sci U S A 98: 11479–11484. 11562485 10.1073/pnas.201394398 PMC58755

[26] Ganal MW, Durstewitz G, Polley A, Berard A, Buckler ES, et al (2011) A large maize (Zea mays L.) SNP genotyping array: development and germplasm genotyping, and genetic mapping to compare with the B73 reference genome. PLoS One 6: e28334. 22174790 10.1371/journal.pone.0028334 PMC3234264

[27] Hubisz MJ, Falush D, Stephens M, Pritchard JK (2009) Inferring weak population structure with the assistance of sample group information. Mol Ecol Resour 9: 1322–1332. 21564903 10.1111/j.1755-0998.2009.02591.x PMC3518025

[28] Yang Z, Zhang E, Li J, Jiang Y, Wang Y, et al (2014) Analyses of sequence polymorphism and haplotype diversity of LEAFY genes revealed post-domestication selection in the Chinese elite maize inbred lines. Mol Biol Rep 41: 1117–1125. 24381105 10.1007/s11033-013-2958-8

[29] Zhang E, Yang Z, Wang Y, Hu Y, Song X, et al (2013) Nucleotide polymorphisms and haplotype diversity of RTCS gene in China elite maize inbred lines. PLoS One 8: e56495. 23437145 10.1371/journal.pone.0056495 PMC3577901

[30] Wang GD, Cheng LG, Fan RX, Irwin DM, Tang SS, et al (2013) Signature of balancing selection at the MC1R gene in Kunming dog populations. PLoS One 8: e55469. 23424634 10.1371/journal.pone.0055469 PMC3570536

[31] Myers S, Bottolo L, Freeman C, McVean G, Donnelly P (2005) A fine-scale map of recombination rates and hotspots across the human genome. Science 310: 321–324. 16224025 10.1126/science.1117196

[32] Jiao Y, Zhao H, Ren L, Song W, Zeng B, et al (2012) Genome-wide genetic changes during modern breeding of maize. Nat Genet 44: 812–815. 22660547 10.1038/ng.2312

[33] Tenaillon MI, Sawkins MC, Long AD, Gaut RL, Doebley JF, et al (2001) Patterns of DNA sequence polymorphism along chromosome 1 of maize (Zea mays ssp. mays L.). Proc Natl Acad Sci U S A 98: 9161–9166. 11470895 10.1073/pnas.151244298 PMC55390

[34] Tan Y, Xing Y, Zhang Q, Sun M, Corke H (2001) Quantitative genetic basis of gelatinization temperature of rice. Cereal chemistry 78: 666–674.

[35] Xu F, Zhang G, Tong C, Sun X, Corke H, et al (2013) Association mapping of starch physicochemical properties with starch biosynthesizing genes in waxy rice (Oryza sativa L.). J Agric Food Chem 61: 10110–10117. 24063600 10.1021/jf4029688

